# Antimicrobial Activities of Chemokines: Not Just a Side-Effect?

**DOI:** 10.3389/fimmu.2012.00213

**Published:** 2012-07-23

**Authors:** Marlene Wolf, Bernhard Moser

**Affiliations:** ^1^Theodor Kocher Institute, University of BernBern, Switzerland; ^2^Institute of Infection and Immunity, School of Medicine, Cardiff UniversityCardiff, UK

**Keywords:** chemokines, antimicrobial peptides, immune surveillance, antimicrobial immunity

## Abstract

The large family of chemoattractant cytokines (chemokines) embraces multiple, in part unrelated functions that go well beyond chemotaxis. Undoubtedly, the control of immune cell migration (chemotaxis) is the single, unifying response mediated by all chemokines, which involves the sequential engagement of chemokine receptors on migrating target cells. However, numerous additional cellular responses are mediated by some (but not all) chemokines, including angiogenesis, tumor cell growth, T-cell co-stimulation, and control of HIV-1 infection. The recently described antimicrobial activity of several chemokines is of particular interest because antimicrobial peptides are thought to provide an essential first-line defense against invading microbes at the extremely large body surfaces of the skin, lungs, and gastrointestinal-urinary tract. Here we summarize the current knowledge about chemokines with antimicrobial activity and discuss their potential contribution to the control of bacterial infections that may take place at the earliest stage of antimicrobial immunity. In the case of homeostatic chemokines with antimicrobial function, such as CXCL14, we propose an immune surveillance function in healthy epithelial tissues characterized by low-level exposure to environmental microbes. Inflammatory chemokines, i.e., chemokines that are produced in tissue cells in response to microbial antigens (such as pathogen-associated molecular patterns) may be more important in orchestrating the cellular arm in antimicrobial immunity.

## Introduction

**Figure 1:**
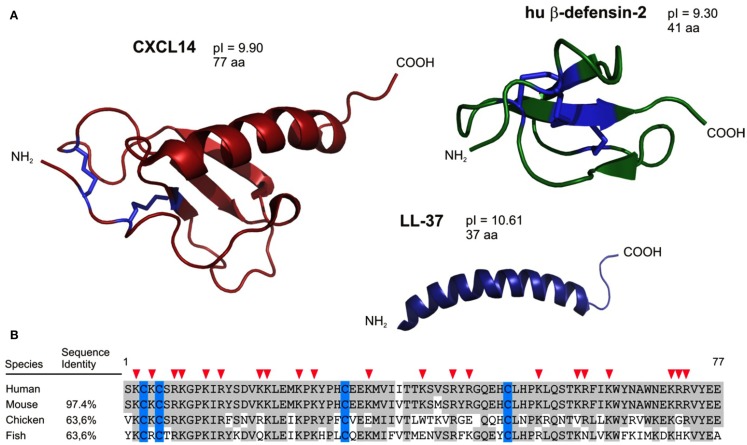
**Structural characteristics predict CXCL14 as an antimicrobial peptide**. **(A)** CXCL14 has structural similarities to both the defensin family (anti-parallel β-sheets) and the cathelicidins (C-terminally located α-helix). In addition, antimicrobial peptides and CXCL14 comprise high *p*I values. **(B)** CXCL14 exhibits high amino acid sequence conservation throughout evolution; 97.4% identity with mouse, 63.6% both with chicken and fish. Red arrow heads denote cationic amino acid residues.

Antimicrobial peptides (AMPs) are components of the innate immune system and are particularly important in the control of cutaneous and intestinal immune defenses (Lai and Gallo, 2009). Underscoring their importance, many AMPs are produced early in phylogenesis, being already present in non-vertebrates, and they exhibit substantial sequence conservation throughout evolution. Initial evidence of host-derived peptides with antimicrobial activities was reported in 1963 in a study describing the discovery of bactericidal basic proteins in the lysosomal granules of polymorphonuclear leukocytes (Zeya and Spitznagel, 1963). Most AMPs are small proteins with less than 50 amino acid residues. More than 800 AMPs have been described since the initial discovery, and among these the defensins and the cathelicidin LL-37 are considered to be the major AMPs present in humans (see reviews, Ganz, 2003; Izadpanah and Gallo, 2005; Cederlund et al., 2011). Defensins and LL-37 are active against a broad spectrum of Gram-negative and Gram-positive bacteria, fungi, and viruses. In line with their antimicrobial activity, they are produced mainly by tissue macrophages and epithelial cells including keratinocytes, Paneth cells, and mucosal epithelial cells but are also present in tissues that are not exposed to exterior microbes. These AMPs are structurally related and positively charged at neutral pH (high *p*I values), and they have the ability to form clusters of hydrophobic and cationic amino acids in aqueous solutions. It is thought that such amphipathic aggregates bind to negatively charged surfaces of microbes leading to membrane disruption and loss of intracellular constituents (Ganz, 2003; Brogden, 2005). In addition to defensins and cathelicidins, there are other AMPs, such as RNAse 7, psoriasin, granulysin, dermcidin, or C-type lectins (Wiesner and Vilcinskas, 2010). Their mechanism of antimicrobial action is not well characterized, but seems to be different from that of the defensins (Mukherjee et al., 2008). More recently, members of the chemokine family were also found to act as AMPs, and one of the first and best characterized “AMP chemokine” is CCL20 (Hoover et al., 2002). Of note, the overall tertiary structure of chemokines defined by disulfide bonds, anti-parallel β-strands, and a C-terminal α-helix is very similar to the one seen in defensins (Figure 1). Even LL-37, the active part of the cathelicidin, shares striking structural similarities with the C-terminal α-helix of chemokines. In addition, similar to defensins and cathelicidins, many chemokines are highly positively charged at neutral pH, which may further explain their antimicrobial activity. Finally, chemokines are also known to form dimers and multimeric aggregates leading to elevated local concentrations (Proudfoot et al., 2003). Of interest, cationic AMPs share functional properties with chemokines, such as chemotactic activity (Yang et al., 1999; Agerberth et al., 2000; De et al., 2000; Wu et al., 2003; Soruri et al., 2007) and were reported to participate in inflammation, wound healing, and adaptive immune responses (Heilborn et al., 2003; Niyonsaba et al., 2007).

The discovery that members of the chemokine family have AMP activity, in addition to their prototype chemoattractant functions, suggests a key role in the control of antimicrobial immunity and merits a more detailed discussion. Simplistically, chemokines are divided into two functional groups: the “homeostatic” chemokines present at distinct locations throughout the body, and “inflammatory” chemokines that are induced locally in response to infection and inflammation (Moser et al., 2004). Members of both groups were shown to display *in vitro* AMP activity, suggesting their involvement not only at the earliest stages of infection but also during the inflammatory phase of antimicrobial immunity. Consequently, one may propose that one function of homeostatic AMP chemokines produced in healthy peripheral tissues is meant to contain microbes in order to prevent the onset of an inflammatory cascade (and associated tissue damage) in response to low dose microbial exposures. Inflammatory AMP chemokines on the other hand act in concert with recruited immune cells in response to substantial microbial infections. Therefore, homeostatic and inflammatory AMP chemokines may target asymptomatic (“unnoticed”) and symptomatic (“inflammatory”) infections, respectively. Before discussing AMP chemokines we will summarize the structure, production, and functions of classical AMPs.

## “Classical” (Non-Chemokine) AMPs

Antimicrobial peptides constitute a primordial immune defense mechanism operating in both vertebrate and invertebrate organisms (Zasloff, 2002). The detailed mechanisms underlying bacterial killing are not fully understood. However, based on their net positive charges, the cationic AMPs are suggested to interact with the negatively charged membranes of bacteria via electrostatic interaction. The amphipathic moieties in AMP aggregates facilitate their insertion into the bacterial membrane, which eventually leads to membrane permeabilization, leakage of ions, and consequently bacterial cell death (Brogden, 2005; Wiesner and Vilcinskas, 2010). In the following section, we briefly discuss the most important “classical” AMPs found in humans. Particularly abundant in humans (and mammals) are the defensins and the cathelicidins, and their impact in immune defense are excellently summarized in a recent review (Lai and Gallo, 2009). The defensins have molecular masses between 3.5 and 6.0 kDa, are positively charged and contain six cysteine residues that form three intramolecular disulfide bridges (Ganz, 2003; Lehrer, 2004; De Smet and Contreras, 2005). On the basis of the size and the disulfide arrangement, the defensins are divided into two subfamilies: the α-defensins and β-defensins. In humans, six different α-defensins and four β-defensins have been identified and characterized. The α-defensins HNP-1 to HNP-4 (originally named *h*uman *n*eutrophil *p*roteins) are stored in azurophilic granules of neutrophils where they constitute upto 50% of the proteins. The α-defensins HD-5 and HD-6 (stands for human defensin) on the other hand, are produced specifically by intestinal Paneth cells (Rehaume and Hancock, 2008). The human β-defensins (hBD)-1 to hBD-4 are mainly synthesized by epithelial cells at diverse body surfaces such as skin, intestine, or trachea (Ganz, 2003). Cathelicidins are synthesized as inactive propeptides with a conserved N-terminal region that is proteolytically removed to liberate the active peptide. In humans, only one cathelicidin is produced, the propeptide hCAP-18 which is enzymatically processed by serine proteases from neutrophil granules (Sorensen et al., 2001) or keratinocytes (Yamasaki et al., 2006) into an active peptide of 37 amino acids (LL-37; Zanetti, 2004). Moreover, LL-37 can be processed to smaller peptides with potent antimicrobial activity such as RK-31 and KS-30 (Murakami et al., 2004). Of note, and in contrast to the “salt-sensitive” defensins, LL-37 was reported to be also active (albeit to a lesser degree) at high sodium chloride concentrations such as frequently encountered in the skin (Travis et al., 2000; Frick et al., 2006). The list of “classical” AMPs includes many other structurally unrelated peptides and proteins such as enzymes, enzyme inhibitors, neuropeptides, or complement-derived peptides (Lai and Gallo, 2009; Wiesner and Vilcinskas, 2010).

Due to constant microbial exposure, skin, and gastrointestinal body surfaces are principal sites of AMP expression. More than 20 AMPs are known to be present in human skin, and the most important of them belong to the molecular families of defensins and cathelicidins as discussed above or are RNases, S100 proteins, or neuropeptides (Schauber and Gallo, 2009). The skin AMPs are either constitutively expressed by epidermal keratinocytes or are induced in response to skin infection or tissue damage. The human cathelicidin proprotein (*hCAP-18*) and precursor of the active LL-37 is not expressed in healthy human skin, but is induced by inflammatory or infectious stimuli (Frohm et al., 1997). Vitamin D3 metabolites, constitutively produced in the skin during exposure to UVB radiation, are also inducers of cathelicidin production (Liu et al., 2006; Schauber and Gallo, 2008). LL-37 shows *in vitro* antimicrobial activity at micromolar concentrations against a wide range of both Gram-negative (*P. aeruginosa*, *S. typhimurium*, *E. coli*) and Gram-positive (*S. aureus*, *S. epidermidis*, and *L. monocytogenes*) bacteria and fungi. Production of defensins in the skin happens both under steady-state and inflammatory conditions. hBD-1 was shown to be constitutively expressed, whereas the expression of hBD-2 and hBD-3 is induced in keratinocytes by pro-inflammatory cytokines and, importantly, by microbes-derived pathogen-associated molecular patterns (Ganz, 2003). Dermcidin, a 47-residue AMP, is constitutively produced by eccrine glands and represents the principal antimicrobial peptide in sweat (Schittek et al., 2001). Importantly, in contrast to most other AMPs, dermcidin is not affected by low pH and increased salt concentrations, conditions that are typically found in sweat (Schittek et al., 2001). A major AMP of healthy human skin, but also of tonsils and pharynx is RNAse 7 (Harder and Schroder, 2002; Zasloff, 2009). RNAse 7 exhibits broad spectrum antimicrobial activity at low micromolar concentrations against both Gram-negative (*E. coli* and *P. aeruginosa)* and Gram-positive (*Propionibacterium acnes*, *S. aureus)* bacteria and the yeast *C. albicans*. Similar to hBD-2 and hBD-3, RNAse 7 expression in primary keratinocytes is induced by pro-inflammatory conditions and microbial stimuli (Harder and Schroder, 2002). Psoriasin, identified as the main “*E. coli* killing factor”, is an 11-kDa protein with preferential expression in the uppermost layer (stratum corneum) of the epidermis (Glaser et al., 2005). Elevated levels of psoriasin were further described in diseased skin, such as atopic dermatitis (AD) and psoriasis (Simanski et al., 2010).

Because several cutaneous AMPs are sensitive to elevated sodium chloride concentrations, it remained speculative whether they play a major role in local immune defense. However, experimental evidence supports a corresponding function since absence or malfunction of cutaneous AMPs are often correlated with skin disease (Lai and Gallo, 2009). For instance, mice deficient in the CRAMP-gene, the mouse homolog of human LL-37, were more susceptible to skin infections caused by group A *Streptococcus*, suffered from a delay in wound healing and also had more severe urinary tract infections than their wild type counterparts (Nizet et al., 2001; Chromek et al., 2006). Mice deficient in β-defensin-1 (mBD-1) showed impaired clearance of *S. aureus* in the lungs and urinary tract compared to wild type (Morrison et al., 2002; Moser et al., 2002). Likewise, altered levels of AMPs appear to play a role in the susceptibility to infections in patients with chronic inflammatory skin disorders. Substantial up-regulation of LL-37 and hBD-2 production was observed in psoriasis (Frohm et al., 1997). In agreement with a role for these peptides in antimicrobial immunity, psoriasis lesions are rarely infected, which is in clear contrast to AD lesions characterized by base-level AMP production and frequent bacterial infections in their excemas (Ong et al., 2002; Nomura et al., 2003). LL-37 production was also found to be upregulated in neonatal skin, which may be viewed as a compensatory mechanism for lack of an effective cellular antimicrobial immune system in newborns (Dorschner et al., 2003). Furthermore, changes in hBD-1 activity have been linked to the lung pathogenesis of cystic fibrosis (CF; Goldman et al., 1997). The pulmonary mucosa of CF patients is often colonized by *P. aeruginosa* which may be neutralized in part by local AMPs. Although AMP knock-down experiments in humans cannot be performed, it is now generally accepted that AMPs contribute to the local antimicrobial immune responses. The emerging view supports the notion whereby inducible AMPs rather than those present in healthy tissues contribute to the clearance of microbes at sites of chronic inflammatory diseases.

In addition to the antimicrobial activity, several AMPs exhibit immunoregulatory properties, including chemotactic activity for immune cells. LL-37 interacts with the formyl peptide receptor like receptor FPRL-1 and mediates chemotaxis of neutrophils, monocytes, and CD4^+^ T-cells (Agerberth et al., 2000; De et al., 2000). Intriguingly, FPRL-1 does not seem to be involved in LL-37 mediated migration of mast cells (Niyonsaba et al., 2002). Moreover, hBD-1, -2, and -3 were reported to bind to CCR6, the receptor for CCL20, and to mediate chemotactic responses in immature dendritic cells and memory T-cells (Yang et al., 1999). However, this finding has been disputed by others (Soruri et al., 2007). hBD-3 was also reported to interact with CXCR4 (Feng et al., 2006), and to be a chemoattractant for monocytes, which do not express CCR6 (Wu et al., 2003). Finally, several reports describe the chemoattractant activity of α-defensins (Yang et al., 2000; Grigat et al., 2007; Rehaume and Hancock, 2008). In addition to immune cell migration, AMPs were also described to affect alternative cellular responses as diverse as cell proliferation, wound healing, angiogenesis, and the release of cytokines and histamine (Bals and Wilson, 2003; Heilborn et al., 2003; Davidson et al., 2004; Niyonsaba et al., 2007; Rehaume and Hancock, 2008).

## Chemokines Acting as AMPs

The first report describing chemokines with antimicrobial function appeared in 2000 (Krijgsveld et al., 2000). The authors identified two antibacterial proteins in platelet granules and showed them to be variants of CXCL7 (NAP-2) and CTAP-3, respectively, that lacked the two C-terminal amino acids (Ala-Asp). Of interest, the intact proteins did not display antimicrobial activity, pointing to the importance of the C-terminal regions as will be discussed in Section “Structure-Function Considerations of Chemokine-AMPs.” Subsequently, additional reports of chemokines with antimicrobial activity appeared in rapid succession, providing evidence that antimicrobial activity is not an exceptional feature of only a few chemokines. Based on structural consideration, i.e., secondary structure similarities between chemokines and defensins (Territo et al., 1989; Chertov et al., 1996; Yang et al., 1999), the non-ELR chemokines CXCL9, CXCL10, and CXCL11 were tested *in vitro* in *E. coli* and *L. monocytogenes* killing assays and found to be potent AMPs (Cole et al., 2001). These authors further demonstrated that CXCL8, CXCL5, CCL2, CCL3, CCL4, CCL5, CX3CL1, and XCL1 were all inactive in the respective *in vitro* assays. Subsequently, J. Oppenheim and colleagues carried out an almost complete screen of human chemokines and reported that, indeed, chemokines with antimicrobial activity are not rare (Yang et al., 2003). Based on unifying structure and electrostatic features, the authors concluded that antimicrobial chemokines share several positively charged surface patches that seemed to be required for antimicrobial activity.

The findings of numerous additional studies that followed these initial reports are summarized in Table 1 (including respective references). Of a total of 45 human chemokines, 23 (10 CXC and 13 CC chemokines) were reported to exhibit antimicrobial activity. It is important to note that several reports remained controversial, i.e., could not be reproduced by other laboratories, which, probably, is due to differences in the selected experimental procedures (bacterial species, bacterial culture conditions, conditions for liquid culture killing and radial diffusion assays, etc.). For example, CXCL6 was found by some groups to be a potent AMP against several Gram-negative and Gram-positive bacteria (Collin et al., 2008; Linge et al., 2008) whereas earlier experiments performed by another group did not support this conclusion (Yang et al., 2003). In contrast to the chemotactic properties of chemokines, which are specific for leukocytes bearing the corresponding chemokine receptors, no specific pattern for the antimicrobial activity has been detected so far. In fact, chemokine-mediated bacterial killing is completely unrelated to chemokine receptor specificity and appears to involve protein structures that are different from chemokine receptor-binding motifs.

**Table 1:** **Antimicrobial activities of chemokines**.

Chemokine	Receptor	Function	Microbial targets (activity)	Reference
**NATURAL CHEMOKINES WITH ANTIMICROBIAL ACTIVITY**				
CXCL1/GROα	CXCR2	Inflammatory	*E. coli* (10 μg/ml: 65–87% killed)	Yang et al. (2003)
CXCL2/GROβ	CXCR2		*S. aureus* (10 μg/ml: 22–39% killed)	
CXCL3/GROγ	CXCR2
CXCL6/GCP-2^1^	CXCR1/R2	Inflammatory	*N. gonorrhoeae* (1 μM: 60% killed)	Collin et al. (2008)
			*E. faecalis* (1 μM: 100% killed)	Linge et al. (2008)
			*S. pyogenes* (MBC^2^_90_: 0.30)
			*S. dysgalactiae* (MBC_90_: 0.28)	
			*S. aureus* (MBC_90_: 0.59)	
			*E. coli* (MBC_90_: 0.46)	
			*P. aeruginosa* (MBC_90_: 0.96)	
CXCL7_1–68_ (=TC-1)^3^	CXCR2		*B. subtilis* (MBC_100_: 0.4–0.7)	Krijgsveld et al. (2000)
PBP^4^_10–92_ (=TC-2)			*S. aureus* (MBC_100_: 6.8–11.0)	
			*E. coli* (MBC_100_: 2.7–3.4)	
CXCL9/MIG	CXCR3	Inflammatory	*E. coli* (MIC^5^: 0.5 (CXCL9), 4.4 (CXCL10), 6.5 (CXCL11)	Cole et al. (2001)
CXCL10/IP-10	CXCR3		*L. monocytogenes* (MIC: 3.6 (CXCL9), 7.0 (CXCL10), 8.0 (CXCL11)	Yang et al. (2003)
CXCL11/I-TAC	CXCR3/R7		*E. coli* (10 μg/ml: 72–100% killed)^6^	Crawford et al. (2009)
			*S. aureus* (10 μg/ml: 69–81% killed)	Crawford et al. (2010)
			*B. anthracis spores* [EC_50_; μg/ml: 6.2 (CXCL9), 7.9 (CXCL10), 28.2 (CXCL11)]	Egesten et al. (2007)
			*S. pyogenes* [EC_50_; μM: 0.022 (CXCL9), 0.17 (CXCL10 and CXCL11)]	
CXCL12/SDF-1	CXCR4/R7	Homeostatic	*E. coli* (10 μg/ml: 86% killed)	Yang et al. (2003)
			*S. aureus* (10 μg/ml: 59% killed)	
CXCL13/BCA-1	CXCR5	Homeostatic	*E. coli* (10 μg/ml: 83% killed)	Yang et al. (2003)
			*S. aureus* (10 μg/ml: 50% killed)	
CXCL14/BRAK	Unknown	Homeostatic	*E. coli* (10 μg/ml: 50% killed)	Yang et al. (2003)
			*S. aureus* (10 μg/ml: 35% killed)	Frick et al. (2011)
			*S. pyogenes* (1 μM: 100% killed)	Maerki et al. (2009)
			*F. magna* (1 μM: 100% killed)	
			*E. coli* (MIC: 0.32)	Maerki et al. (2009)
			*S. coag.neg*. (MIC: 1.02)	
			*S. aureus* (MIC: 5.17)	
			*Propionibact*. (MIC: 1.16)	
			*C. albicans* (MIC: 0.56)	
XCL1/lymphotactin^7^	XCR1		*E. coli* (10 μg/ml: 100% killed)	Yang et al. (2003)
			*S. aureus* (10 μg/ml: 47% killed)	
CCL1/I-309	CCR8	Homeostatic	*E. coli* (10 μg/ml: 88% killed)	Yang et al. (2003)
			*S. aureus* (10 μg/ml: 37% killed)	
CCL2/MCP-1^7^	CCR2/R4	Inflammatory	*E. coli* (LD_90_: 30 μg/ml)	Hoover et al. (2003)
CCL11/Eotaxin	CCR3	Inflammatory	*E. coli* (10 μg/ml: 66% killed)	Yang et al. (2003)
			*S. aureus* (10 μg/ml: 49% killed)	
CCL13/MCP-4^8^	CCR2/R3	Inflammatory	*E. coli* (10 μg/ml: 76% killed)	Yang et al. (2003), Martinez-Becerra et al. (2007)
CCL14	CCR1	Inflammatory	*E. coli* (1 μM: 60% killed)	Kotarsky et al. (2010)
CCL15	CCR1/R3	
CCL17/TARC	CCR4	Inflammatory	*E. coli* (10 μg/ml: 59% killed)	Yang et al. (2003)
			*S. aureus* (10 μg/ml: 46% killed)	
CCL18/PARC		Homeostatic	*E. coli* (10 μg/ml: 100% killed)	Yang et al. (2003)
			*S. aureus* (10 μg/ml: 69% killed)	
CCL19/ELC^8^	CCR7	Homeostatic	*E. coli* (10 μg/ml: 82% killed)	Yang et al. (2003)
CCL20/LARC	CCR6	Inflammatory	*E. coli* (LD_50_: 0.4 μg/ml,)	Yang et al. (2003)
			*S. aureus* (LD_50_: 10 μg/ml)	Hoover et al. (2002)
			*C. albicans* (LD_50_: 25 μg/ml)	Kim et al. (2007)
			*S. pyogenes* (LD_50_: 0.2 μg/ml)	
			*E. faecium* (LD_50_: 4.5 μg/ml)	
			*P. aeruginosa* (LD_50_: 1.1 μg/ml)	
			*M. catarrhalis* (LD_50_: 0.9 μg/ml)	
			*Vaccinia virus*	
CCL21/SLC	CCR7	Homeostatic	*E. coli* (10 μg/ml: 45% killed)	Yang et al. (2003)
			*S. aureus* (10 μg/ml: 75% killed)	
CCL22/MDC	CCR4	Inflammatory	*E. coli* (10 μg/ml: 100% killed)	Yang et al. (2003)
			*S. aureus* (10 μg/ml: 74% killed)	
CCL25/TECK	CCR9	Homeostatic	*E. coli* (10 μg/ml: 68% killed)	Yang et al. (2003)
			*S. aureus* (10 μg/ml: 48% killed)	
CCL28/MEC	CCR3/R10	Inflammatory	*C. albicans* (IC_50_: 0.7 μM)	Hieshima et al. (2003)
			*P. aeruginosa* (IC_50_: 0.4 μM)	
			*S. mutans* (IC_50_: 1.7 μM)	
			*S. aureus* (IC_50_: 0.9 μM)	
			*S. pyogenes* (IC_50_: 3.0 μM)	
			*K. pneumoniae* (IC_50_: 0.3 μM)	
**CHEMOKINES WITHOUT ANTIMICROBIAL ACTIVITY**^9^				
CXCL5/ENA-78	CXCR2	Inflammatory	*E. coli*, *S. aureus*, *C. albicans*	Yang et al. (2003)
CXCL6/GCP-2^10^	CXCR1/R2			Cole et al. (2001)
CXCL7/NAP-2	CXCR2			Maerki et al. (2009)
CXCL8/IL-8	CXCR1/R2	
CX3CL1/Fractalkine	CX3CR1/R2	
CCL2/MCP-1^11^	CCR2/4	Inflammatory	*E. coli*, *S. aureus*, *B. anthracis spores*, *P. aeruginosa*, *S. mutans*	Yang et al. (2003)
CCL3/MIP-1α	CCR1/R4/R5			Cole et al. (2001)
CCL4//MIP-1β	CCR5			Crawford et al. (2009)
CCL5/RANTES	CCR1/R3/R4/R5			Crawford et al. (2010)
CCL7/MCP-3	CCR1/R2/R3			Hieshima et al. (2003)
CCL16/HCC-4	CCR1			Liu and Wilson (2010)
CCL27/CTACK	CCR10	
**CHEMOKINE-DERIVED PEPTIDES WITH ANTIMICROBIAL ACTIVITY**				
CXCL7_50–70_^12^			*B. subtilis* (LD_99.9_ > 120 μM)	Nguyen et al. (2010)
CXCL7_50–68_ (=TC-1_50–68_)			*S. aureus* (LD_99.9_ > 120 μM)	
			*E. coli* (LD_99.9_ > 120 μM)	
CXCL8_80–99_^13^			*E. coli* (LD_99.9_ > 120 μM)	Nguyen et al. (2010)
CXCL8_81–99_			*K. pneumoniae*	Bjorstad et al. (2005)
CXCL8_53–72_			*S. pyogenes*	
			*H. pylori*	
			*E. enterica MR10*	
			*B. subtilis* (LD_99.9_ > 120 μM)	
			*S. aureus* (LD_99.9_ > 120 μM)	
CXCL9_79–105_			*S. pyogenes* (ED_50_: 0.02 μM)	Egesten et al. (2007)
CCL13_57–75_ (=CDAP-4)			*E. coli* (6.8 μM: 47% killed)	Martinez-Becerra et al. (2007)
			*P. aeruginosa* (6.8 μM: 91% killed)	
			*S. typhi* (6.8 μM: 84% killed)	
			*S. typhimurium* (6.8 μM: 91% killed)	
			*K. pneumonia* (6.8 μM: 47% killed)	
CCL20_51–70_			*E. coli* (MIC: 12.5 μg/ml; LD_99.9_ ≤ 0.45 μM)	Nguyen et al. (2010)
CCL20_59–70_			*S. aureus* (MIC: 63 μg/ml; LD_99.9_: 1.9 μM)	Chan et al. (2008)
			*B. subtilis* (LD_99.9_ ≤ 0.45 μM)	Hasan et al. (2006)
			*E. coli* (LD_50_: 3.2 μg/ml); (LD_99.9_: 30 μM)	
			*B. subtilis* (LD_99.9_: 15 μM)	
CCL28_77–105_^14^			*S. aureus* (IC_50_: 7.0 μM)	Hieshima et al. (2003)
			*C.albicans* (IC_50_: 1.6 μM)	Liu and Wilson (2010)
			*P. aeruginosa*	

*^1^No activity with *C. albicans*; inactive in Yang et al. (2003)*.

*^2^MBC_90_, *minimal bactericidal concentration to kill 90% of bacteria*, μM*.

*^3^Full-size CXCL7_1–70_ has no activity*.

*^4^Platelet Basic Protein*.

*^5^MIC, minimal inhibitory concentration, μM*.

*^6^Crawford et al. (2010) shows *in vivo* evidence*.

*^7^Inactive in Cole et al. (2001)*.

*^8^No activity with *S. aureus**.

*^9^Tested, but not found to be active*.

*^10^CXCL6 is reported as active in Yang et al. (2003), Collin et al. (2008)*.

*^11^CCL2 is reported as active in Hoover et al. (2003)*.

*^12^Full-size CXCL7_1–70_ has no activity*.

*^13^Full-size CXCL8 has no activity*.

*^14^Positively charged C-terminus essential, but not sufficient*.

Most studies exploring antimicrobial activities of chemokines have been limited to *in vitro* investigations and very few data conclusively support a role as AMPs in *in vivo* infection models. One of the difficulties associated with *in vivo* studies is the fact that chemokines induce the recruitment of leukocytes, whose cellular responses against microbes may obscure their *bonafide* AMP function. Importantly, in most *in vitro* studies chemotactic responses were elicited at >10-fold lower chemokine concentrations than what were required for AMP activity. *In vivo* relevance of the importance of AMPs in host defense however has been demonstrated with the model of *B. anthracis* infection. In a first study, Crawford et al. (2009) have shown that germination of *B. anthracis* spores can be inhibited by CXCL10. They established a correlation between higher levels of CXCL9, CXCL10, and CXCL11 in the lungs of C57BL/6 mice and the resistance of these mice to respiratory *B. anthracis* infection and showed that A/J mice whose lungs expressed considerably lower levels of these chemokines were highly susceptible to *B. anthracis* infection. Further investigations supported these data by showing that C57BL/6 mice pretreated with CXCL9-, CXCL10-, or CXCL11-neutralizing antibodies were more susceptible to *B. anthracis* infection and that this effect was independent of the chemokine receptor CXCR3 (Crawford et al., 2010).

## Structure-Function Considerations of Chemokine-AMPs

As already briefly mentioned, structural analysis of chemokines and defensins revealed that these two peptide families share structural motifs that may explain their common AMP activities. Most of the structure-function studies involved the comparison between the best described AMP chemokine, CCL20 with hBD-1 and hBD-2 (Hoover et al., 2002; Chan et al., 2008). The homology of these peptides, however, does not lie in the amino acid sequence but is confined to structural features such as cationic residues and amphipathic stretches that are frequent in the COOH-terminal α-helices of the classical AMPs. Peptides with cationic residues and amphipathic stretches have been shown to adopt a helical structure in a membrane mimetic solvent, for example in a 2,2,2-trifluoroethanol/water mixture (Nguyen et al., 2010). In some cases the AMP activity of the C-terminus is hidden by intramolecular interactions, which may neutralize the positively charged C-terminal α-helix (Nguyen et al., 2010). Intramolecular interaction may also explain why C-terminal peptides but not the intact molecule of CXCL8 displays antimicrobial activity (Bjorstad et al., 2005). This also suggests that proteolytic processing of some mature chemokines may be required to convert them into potent AMPs. It is noteworthy that at present we have no idea about the type of protease(s) responsible for converting inflammatory chemokines into AMPs. By contrast, two recent studies demonstrate that bacterial proteases degraded chemokines into inactive peptides (Egesten et al., 2009; Frick et al., 2011), thereby revealing a strategy of pathogens to avoid innate immune mechanism(s) of the host. Such escape mechanisms of bacteria are also known to inactivate classical AMPs, such as LL-37 and dermcidin (Lai and Gallo, 2009). Interestingly, the M1 strain of *S. pyogenes* secretes the protein “Streptococcal Inhibitor of Complement” (*SIC*) that was shown to block the AMP activity of CXCL9 (Egesten et al., 2007). It appears that the negatively charged *SIC* competes with glycosaminoglycans (GAGs) for binding to the positively charged CXCL9, suggesting an important role of CXCL9-GAGs interactions on epithelial surfaces for controlling antimicrobial activity. Furthermore, as reported for CXCL9 (Egesten et al., 2011), plasma proteins may also indirectly inhibit antimicrobial activity by binding to the cell wall proteins of bacteria, thereby interfering with the binding of AMPs.

Initial structure-function studies assumed that the disulfide bonds are essential for antimicrobial activity. However, recent work revealed that hBD-1, known as an AMP with low antimicrobial activity, turned into a potent AMP following reduction of its disulfide bonds (Schroeder et al., 2011). hBD-1 has restricted specificity for Gram-negative bacteria, but acquired broad spectrum antimicrobial activity against anaerobic Gram-positive bacteria and *C. albicans*, upon reduction of the disulfide bonds. The importance of disulfide bonds does not apply to all AMPs because mutations of cysteine residues in CCL28 (Liu and Wilson, 2010) or hBD-3 (Wu et al., 2003), did not affect their AMP.

Today, the AMP activity of several chemokines is firmly established although the underlying mechanism(s) remain ill defined. On the basis of structural considerations (Yang et al., 2003), most laboratories have assumed that cationic chemokines attack bacterial membranes similar to cationic and amphipathic defensins. In agreement, several reports demonstrate that chemokines bind to bacteria and induce leakage of intracellular contents, as shown for CXCL14 (Frick et al., 2011) and CXCL6/GCP-2 (Linge et al., 2008). Furthermore, full-length CXCL6 was compared with deletion variants composed of either the C-terminal 19 amino acids or the NH_2_-terminal 50 amino acids. Both variant peptides displayed lower antimicrobial activity than the full-length CXCL6. However, the NH_2_-terminal peptide was the more potent of the two variant peptides and was able to induce in sodium chloride-sensitive fashion membrane damage in carboxyfluorescein-loaded liposomes. In contrast, other studies concluded that the membrane attack is not the principal killing mechanism. For instance, the CXCL7 derivatives TC-1 or TC-2 inhibited several bacterial species but did not act on bacterial membranes, as evidenced by lack of changes in membrane potential (Krijgsveld et al., 2000). Also, CCL6 induced only partial membrane depolarization, a further sign that alternative mechanisms may contribute to the observed antimicrobial activity (Kotarsky et al., 2010).

Comparing hydrophobicity regions in ELR chemokines with those of non-ELR chemokines, Cole et al. (2001) reported that most notable differences reside in the C-terminal helices, which are more polar and positively charged in non-ELR chemokines. Accordingly, the non-ELR chemokines CXCL9, CXCL10, and CXCL11 were found to display antimicrobial activity whereas the ELR chemokines CXCL5 and CXCL8 failed to do so. Along the same line, the last five C-terminal amino acids (Arg-Lys-Asp-Arg-Lys) in CCL28 were found to be essential for bacterial killing (Liu and Wilson, 2010). Mutant chemokines missing these five amino acids or variants where the arginine was replaced by the neutral alanine or the negatively charged aspartate had diminished AMP activity. Moreover, these authors demonstrated that the C-terminal region by itself was inactive and that full activity depended on NH_2_-terminal regions (either supplied by CCL28 or by CCL5 in chimeric chemokine constructs). Of note, the structures responsible for antimicrobial activity differ from those involved in controlling leukocyte chemotaxis (Clark-Lewis et al., 1994). In conclusion, more work is required to fully understand the structures involved in bacterial killing, which may lead to engineered chemokine variants with improved antimicrobial activity suitable for clinical use.

## Human CXCL14: An Example of a Broad Spectrum Antimicrobial Chemokine

Our group has a special interest in CXCL14, one of the most recent chemokines (Hromas et al., 1999; Frederick et al., 2000; Sleeman et al., 2000). This non-ELR chemokine is unusual for three reasons. First, it is the least understood chemokine, owing at least in part to the fact that its receptor is still unknown. Second, CXCL14 has an unusual primary structure (Figure 1); more precisely, its NH_2_-terminal amino acid sequence preceding the first Cys residue consists of only two amino acids (Ser-Lys). Normally, NH_2_-terminal regions in chemokines have five or more residues, which are known to be important for receptor interaction (Clark-Lewis et al., 1994). Predicted differences from “classical” chemokines with respect to receptor-binding may explain, in part, our current rudimentary knowledge about the role(s) played by CXCL14 in physiological processes (see below). Third, the primary amino acid sequence of CXCL14 is highly conserved across vertebrates as diverse as fish, birds, and mammals, suggesting an important (non-redundant) function (Figure 1). For instance, human and mouse CXCL14 differ only in two conservative amino acid substitutions (Ile_36_ → Val_36_ and Val_41_ → Met_41_).

What is known about the function of human CXCL14? Our studies revealed that CXCL14 is a low potent chemoattractant for human blood monocytes (Kurth et al., 2001) whereas other groups provided evidence for a role in DC migration (Sleeman et al., 2000; Shellenberger et al., 2004; Shurin et al., 2005; Salogni et al., 2009). Numerous reports have implicated CXCL14 in cancer but findings disagree whether this chemokines displays anti-tumor as opposed to tumor-promoting activity (Frederick et al., 2000; Shurin et al., 2005; Ozawa et al., 2006; Wente et al., 2008; Augsten et al., 2009). A hallmark of CXCL14 is its constitutive expression at high levels in many epithelial tissues, most notably in the skin and gastrointestinal tract (Schaerli et al., 2005; Meuter and Moser, 2008; Maerki et al., 2009). Recent reports describe substantial expression of CXCL14 in taste bunds of human and mouse tongues (Hevezi et al., 2009). CXCL14 protein was also detected in rat and murine neurons (Schmid et al., 2009; Yamamoto et al., 2011) and recently, was proposed to be involved in inhibiting the effects of synaptically released GABA (Banisadr et al., 2011). Of interest, some differences exist between humans and mice since the lung tissue of mouse but not human stained positive for CXCL14 (Meuter and Moser, 2008). Additional CXCL14 mRNA-positive tissues include ovary, brain, kidney, and trachea. CXCL14 is produced highest in keratinocytes and other epithelial cells of mucosal tissues as well as associated macrophages. CXCL14 is not produced by leukocytes (other than tissue macrophages) and is not found in secondary lymphoid organs, arguing for a distinct (perhaps unique) function in immunity. Current evidence supports the view that CXCL14 is a homeostatic as opposed to inflammatory chemokine (Moser et al., 2004). In fact, it appears that epidermal expression of CXCL14 is down-modulated under inflammatory conditions, arguing for a role in tissue homeostasis rather than inflammation (Maerki et al., 2009; Frick et al., 2011). Thus far, work with CXCL14-deficient mice has not greatly advanced our understanding about the function of CXCL14 (Meuter et al., 2007; Nara et al., 2007; Tanegashima et al., 2010).

**Figure 2:**
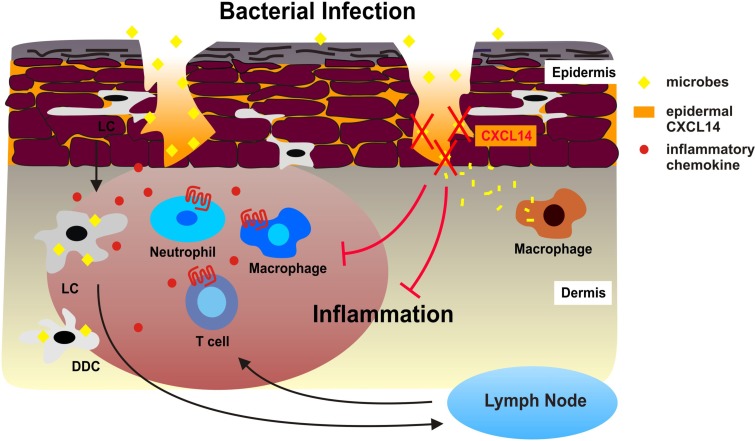
**Antimicrobial activity of CXCL14 during superficial injuries in human skin**. During bacterial infections, Langerhans cells (LC), dermal DC (DDC), and other sentinel cells of the skin produce inflammatory chemokines in response to microbial stimuli, which immediately attract neutrophil and monocytes/macrophages from circulation. DC also take up and process microbes and emigrate to draining lymph nodes as a consequence of microbe-induced DC maturation. Subsequent effector T cells are recruited to the site of microbial infection. The combination of innate and adaptive immune responses may exacerbate tissue inflammation. We propose that the high levels of CXCL14 in the epidermis neutralize microbes that enter the skin during micro-injuries and dermal macrophages then clean-up bacterial debris. This instant antimicrobial system safeguards the skin from excessive cellular immune responses, thereby contributing to the maintenance of tissue integrity.

We have reported a severe breeding defect that may have resulted from disturbed antimicrobial immunity in placenta tissue or from abnormal trophoblast migration (Meuter et al., 2007; Kuang et al., 2009). This behavior together with CXCL14 expression in taste buds of the tongue fully agree with a role of CXCL14 in antimicrobial immunity (Hevezi et al., 2009). Indeed, our *in vitro* studies have revealed that CXCL14 has broad spectrum antimicrobial activity for Gram-positive as well as Gram-negative bacteria, including skin commensals as well as frequent pathogens (Maerki et al., 2009; Table 1). CXCL14 also killed the yeast *C. albicans*. CXCL14 is considerably larger than non-chemokine-AMPs; however, it shares several structural features including a high density of positive charges at physiological pH as well as a core-structure consisting of three anti-parallel β-strands reminiscent of the β-defensin “fold” and a C-terminal α-helix that is typical for LL-37 (Figure 1). The antimicrobial activity in CXCL14 is sensitive to tissue proteases, suggesting that the high turn-over in healthy tissues needs to be maintained by steady-state *de novo* protein synthesis. CXCL14 production is not observed in primary keratinocyte cultures but is abundant in epidermis as well as artificial epidermal equivalents, pointing to regulation by epidermal differentiation factors (Schaerli et al., 2005). In support of a homeostatic role, inflammatory stimuli are inhibitory and lead to down-regulation of CXCL14 expression (Maerki et al., 2009; Frick et al., 2011). Finally, it was recently shown that UVB radiation induced CXCL14 expression in cultured squamous carcinoma cells (Ozawa et al., 2010) that may involve Rho family member RhoBTB2 (McKinnon et al., 2008) and transcription factor AP-1 (Pelicano et al., 2009). Still, our knowledge about the factors controlling CXCL14 expression in healthy epithelia and tumors is rudimentary. The model in Figure 2 summarizes our own work with cutaneous CXCL14 but may be extended to CXCL14 at other locations with prominent exposure to environmental microbes (tongue, respiratory, gastrointestinal, and genital tracts).

We propose that CXCL14 contributes to the tonic antimicrobial climate made up by physical (cell impermeable epithelia) and chemical (salt, pH, lipids) factors as well as secreted AMPs within the outermost layer of the skin (epidermis). The antimicrobial activity of CXCL14 may be particularly instrumental in the containment of “steady-state” infections, i.e., non-symptomatic infections that occur during micro-injuries of the skin. These frequent events remain unnoticed and, thus, do not cause inflammation. This homeostatic role of CXCL14 may be supported by other local AMPs if they are present at concentrations sufficient for microbial killing. CXCL14 probably does not play a role in “symptomatic” infections marked by local inflammation where inducible factors, including inflammatory cytokines/chemokines and inducible defensins, take over. As mentioned above, an appropriate model for testing the *in vivo* AMP activity of CXCL14 needs to be developed.

## Discussion/Conclusion

What can we learn from the substantial body of literature dealing with AMP chemokines? First, some but not all chemokines have robust antimicrobial activity, at least during *in vitro* tests. AMP chemokines are cationic (*p*I > 9.0) and have hydrophobic pockets and, therefore, it is reasonable to assume that they interact with bacterial membranes. How AMPs (including AMP chemokines) are able to discriminate between membranes of bacteria with those of the host cells (epithelial tissue cells) is not completely understood. Selectivity for distinct lipid compositions may be a possibility (Lai and Gallo, 2009), and membrane polarity (or asymmetry) may also play a role. Second, chemokines are primarily chemoattractant proteins, and it is not clear whether the observed antimicrobial activities are secondary to the control of leukocyte migration. For instance, the relevance of antimicrobial activity of inflammatory chemokines may be less important because they are produced by local tissue cells in response to infectious microbes that have reached numbers large enough for triggering the inflammatory cytokine cascade. At the inflammatory stage (the stage at which inflammatory chemokines are produced) chemokine-mediated recruitment of phagocytes (neutrophils) and, eventually, lymphocytes may be far more relevant to infection control than direct microbial killing. Furthermore, AMP chemokines that control adaptive immune processes, such as CXCL9, CXCL10, and CXCL11, are frequently produced late in infections and, thus, may be less relevant to microbial killing at the onset of infection.

We propose that the “ideal” AMP chemokine is one that is already present in healthy tissues or rapidly produced by local tissue cells in response to microbes. We believe that CXCL14 fits this bill since it is continuously produced at the right locations (body-environment interfaces). Similar to inducible defensins, neutrophil-, and perhaps monocyte-specific chemokines may also contribute to the antimicrobial climate because of their rapid production during infection. As mentioned above, our model of AMP chemokines needs to be tested in appropriate infection models, which will be one of the main tasks in future research.

## Conflict of Interest Statement

The authors declare that the research was conducted in the absence of any commercial or financial relationships that could be construed as a potential conflict of interest.
